# Do Antiepileptic Drugs Change the Levels of Arginine Derivatives in Epileptic Children Treated with Polytherapy? The Results of a Case–Control Study

**DOI:** 10.3390/children9111709

**Published:** 2022-11-08

**Authors:** Beata Sarecka-Hujar, Izabela Szołtysek-Bołdys, Ilona Kopyta

**Affiliations:** 1 Department of Basic Biomedical Science, Faculty of Pharmaceutical Sciences in Sosnowiec, Medical University of Silesia in Katowice, 41-200 Sosnowiec, Poland; 2 Department of General and Inorganic Chemistry, Faculty of Pharmaceutical Sciences in Sosnowiec, Medical University of Silesia in Katowice, 41-200 Sosnowiec, Poland; 3 Department of Pediatric Neurology, Faculty of Medical Sciences in Katowice, Medical University of Silesia in Katowice, Katowice, 40-752 Katowice, Poland

**Keywords:** epilepsy, children, ADMA, SDMA, homoarginine, arginine derivatives

## Abstract

Previously, a relation between therapy with antiepileptic drugs (AEDs) and the levels of biochemical parameters was observed in adult patients suffering from epilepsy. Among these biochemical factors, arginine derivatives are often analyzed, i.e., asymmetric dimethylarginine (ADMA), symmetric dimethylarginine (SDMA), and homoarginine (hArg) as they may be linked with increased risk for cardiovascular disease (CVD). Since the levels of arginine derivatives may increase during therapy, and the treatment of epilepsy often lasts many years, patients may experience CVD faster. The aim of the present study was to analyze the levels of arginine derivatives in children with epilepsy who were treated with multiple AEDs to answer the question whether pediatric patients may be at increased risk of CVD in the future. We prospectively analyzed 21 children suffering from epilepsy who took ≥2 AEDs for at least 6 months and 22 children without epilepsy (reference group). The levels of the arginine derivatives, e.g., ADMA, SDMA, and hArg, were determined in the blood serum using the HPLC method. No differences in both the mean levels of ADMA and SDMA, as well as in the mean values of the arginine derivative ratios, were observed between the groups. The tendency toward a lower level of hArg was found in epileptic patients more than in the reference group (*p* = 0.091). Epileptic children receiving three or more AEDs had significantly lower concentrations of hArg and values of the hArg/ADMA ratio than the reference group (*p* = 0.023 and *p* = 0.006, respectively). In turn, the mean hArg/ADMA ratio was lower in children receiving three or more AEDs compared to children receiving two AEDs (*p* = 0.002). There was also a positive correlation between the hArg and ADMA concentrations in children with epilepsy taking two AEDs; the higher the level of hArg, the greater the level of ADMA on average (r = 0.650, *p* = 0.022). Taking three or more AEDs by epileptic children resulted in lower levels of both hArg and the value of the hArg/ADMA ratio.

## 1. Introduction

Worldwide, millions of people suffer from epileptic seizures. Based on numerous data, the prevalence of epilepsy in children can be estimated in the range 41–187/100,000 [1]. About 60% of them have seizures that can be controlled using one antiepileptic drug (AED), while others require polytherapy [2,3]. On the contrary, among pediatric patients below 12 years of age from India, only 2.8% of the patients (4 out of 142) were on polytherapy [4]. The proportion of patients on monotherapy versus patients treated on polytherapy may vary depending on the age of the patients. As previously reported, polytherapy with more than two AEDs was over two-fold more often in German children and adolescents than in patients over 65 years of age, which may indicate the more difficult-to-treat epilepsy among children [2].

Two types of AEDs may be distinguished, i.e., drugs inducing or inhibiting cytochrome P450 (CYP) isoenzymes and drugs that have no effect on CYP. In the first group of drugs, carbamazepine (CBZ), phenobarbital (PB), phenytoin (PHT), or primidone induce CYP isoenzymes, while valproate (VPA) inhibits them. In the second group of drugs, gabapentin, levetiracetam (LEV), vigabatrin (VGB), or topiramate (TPM) are included. One of the new generation AEDs, oxcarbazepine (OXC), is a weak inhibitor of P-450 (CYP2C19). OXC may increase the plasma concentrations of other AEDs, which are simultaneously used, and in consequence, it may increase their activity or toxicity [5].

In adult epileptic patients, therapy with AEDs may increase the concentrations of certain biochemical parameters that may be linked to a higher risk for cardiovascular disease (CVD). Among these parameters, new, nonclassical risk factors for atherosclerosis were reported, i.e., arginine derivatives: asymmetric dimethylarginine (ADMA), symmetric dimethylarginine (SDMA), and homoarginine (hArg) [6,7,8,9]. The ADMA level is suggested to be a better indicator of endothelial dysfunction than the serum homocysteine level since it is less sensitive to changes, such as the fasting status or physical activity [7]. Earlier, in the general adult population, the arginine derivatives were linked with impaired CVD outcomes and mortality in patients at risk. However, these associations were opposite one another, i.e., low-circulating hArg, but high concentrations of ADMA and SDMA increased the CVD risk [10,11,12]. Most recently, in the study by Mokhaneli et al. [13], elevated levels of L-homoarginine were correlated to reduce the risk of 10-year cardiovascular and all-cause mortality.

Since the duration of treatment with AEDs often lasts many years and significantly accelerates the process of atherosclerosis, patients may experience cardiovascular events faster. ADMA is an inhibitor of nitric oxide synthase (NOS). When the ADMA concentration increases, the bioavailability of nitric oxide (NO) decreases. ADMA may promote the occurrence of epileptic seizures since it induces changes in the metabolism of arginine, which is a precursor of excitatory (glutamate) and inhibitory neurotransmitters (γ-aminobutyric acid). However, data regarding the association between arginine derivatives and epilepsy are most often contradictory both in adults and children [6,7,8,9]. In the pediatric population, some studies indicated that long usage of AEDs has an impact on the ADMA levels, while other studies did not support such observations [7,9].

Due to the low amount of evidence on the concentrations of arginine derivatives in pediatric epilepsy, we try to compare the levels of ADMA, SDMA, and hARg between children suffering from epilepsy and receiving multiple AEDs for at least 6 months and children with no epilepsy. According to our knowledge, the present evaluation of the SDMA and hArg levels is the first such analysis in the pediatric population.

## 2. Materials and Methods

### 2.1. Study Groups

This study is the second part of a bigger research project analyzing the influence of AEDs on the biochemical parameters in children suffering from epilepsy. In the first part of the project, analyses of the lipid parameters, as well as concentrations of selected aminothiols (i.e., homocysteine, cysteine, and glutathione), were performed [14].

In the present study, 43 children were prospectively recruited by the pediatric neurologist (IK) at the Department of Pediatric Neurology, the Medical University of Silesia in Katowice (Poland) in 2020–2021. Among the recruited children, two study groups were distinguished i.e., an epilepsy group treated with AEDs for at least 6 months (*n* = 21) and a reference group of patients who received no pharmacological treatment with AEDs (*n* = 22). The inclusion/exclusion criteria were described previously [14].

The present study was evaluated by the Ethics Committee of the Medical University of Silesia in Katowice, Poland, and received approval for execution (no. of approval: PCN/0022/KB1/43/20). Each patient was recruited after obtaining written informed consent from the parent.

### 2.2. Analysis of Arginine Derivatives

The concentrations of the arginine derivatives were assessed in the blood serum. Biological samples of antecubital venous blood were collected after 12 h of fasting, and then, the samples were centrifuged within 2 h after being drawn.

The mentioned parameters were determined simultaneously using high-performance liquid chromatography (HPLC) according to the method described by Teerlink et al. [15]: 200 µL of serum were mixed with 100 µL of internal standard solution and 700 µL of PBS. The samples were purified by solid-phase extraction using Waters, Oasis MCX (30 mg) SPE columns. The test substances were eluted from the column with 1.0 mL of concentrated ammonia/water/methanol mixed in a 10:40:50 volumetric ratio. The resulting eluate was evaporated under a vacuum. The dry residue was dissolved in 100 µL of water and derivatized by adding 100 µL of OPA solution. The mixture was shaken vigorously for 1 min. Then, 50 µL of the solution was applied to Waters Symmetry columns C18, 150 × 3.9 mm I.D. The chromatographic separation was carried out under isocratic conditions using 100% phase A (50 mM phosphate buffer containing 8.7% acetonitrile, pH 6.5). An eluent system of 50% phase A and 50% phase B (acetonitrile/water 50:50 *v/v*) was used between 20 and 27 min and, from 28 min, went back to 100% phase A and conditioned the column for another 7 min. Separation and detection were performed at room temperature using a fluorescence detector (excitation 340 nm; emission 455 nm). To separate the overlapping ADMA and SDMA peaks, the control program adopted the nonlinear Marquardt least squares method and the modified Gaussian exponential model as the chromatographic peak model.

All samples were measured twice.

In addition, on the basis of obtained values of arginine derivatives, the ADMA/SDMA, hArg/ADMA, and hArg/SDMA ratios were calculated.

### 2.3. Statistical Analyses

For statistical analyses, STATISTICA 13.0 software (STATSOFT; Statistica, Tulsa, OK, USA) was used. The mean values (M) and the standard deviations (SD) were estimated for the continuous variables. The normality of the data was verified with the Shapiro–Wilk test. Since there were small sizes of the analyzed groups/subgroups of patients, comparisons of the quantitative data were performed using the nonparametric Mann–Whitney *U* test throughout the study. In addition, Pearson’s correlation coefficients between arginine derivatives were estimated. The result was considered as statistically significant when the *p*-value was below 0.05.

## 3. Results

### 3.1. General Characteristics of the Study Groups

The mean age of the analyzed cases, as well as the distribution of girls and boys, were comparable between the two groups (Table 1).

The median duration of the treatment with AEDs was 2.75 years (from 0.5 to 15.5 years). Only one analyzed child with epilepsy had the minimum required time of epilepsy treatment. The frequency of specific combinations of AEDs administered to epileptic children as well as the presence of types of seizures at both onset and follow-up in analyzed epileptic children is shown in Table 2. The etiology of epilepsy in recruited patients was classified according to Scheffer et al. [16] as structural (in 4 children, 19.05%), genetic (in 2 children, 9.52%; Prader–Willi syndrome, Dravet syndrome), and unknown (in 15 children, 71.43%).

The largest percentage of children took a combination of two drugs—VPA and LEV (28.57%), then—VPA, CLB and LEV, LTG (9.52% each). Each of the remained AEs combinations was taken by only one child (Table 2). In the case of the morphology of seizures, generalized seizures, polymorphic seizures, and myoclonic seizures occurred most frequently (66.67%, 23.81, and 23.81, respectively).

### 3.2. Concentrations of Arginine Derivatives in the Analyzed Groups

Table 3 demonstrates the average concentrations of arginine derivatives in children having epilepsy and children epilepsy-free. No differences in both mean levels of ADMA and SDMA, as well as in the mean values of the arginine derivative ratios, were found between the groups. The tendency to a lower level of hArg was found in epileptic patients compared to the reference group (*p* = 0.091).

We did not observe differences in levels of analyzed parameters between girls and boys from the control group. However, in the group of epileptic children, the mean level of SDMA was slightly higher in girls than in boys (0.74 vs. 0.58) but the difference was close to the bound of significance (*p* = 0.089).

### 3.3. Concentrations of Arginine Derivatives Depending to the Number of AEDs

Due to the number of AEDs taken, we have divided the whole epilepsy group into two subgroups: subgroup 1—children receiving two AEDs and subgroup 2—children receiving three or more AEDs. Table 4 presents the average concentrations of the arginine derivatives in subgroups depending on the number of AEDs.

There were significant differences in mean concentrations of hARg, as well as the hArg/ADMA ratio between the three analyzed groups. Post-hoc analysis revealed that epileptic children receiving three or more AEDs presented significantly lower levels of hArg and value of the hArg/ADMA ratio than controls (*p* = 0.023 and *p* = 0.006, respectively). In turn, the mean hArg/ADMA ratio was lower in children receiving three or more AEDs compared to children receiving two AEDs (*p* = 0.002). In turn, there was a tendency to lower the mean level of hArg in children receiving three or more AEDs compared to children receiving two AEDs (*p* = 0.089).

### 3.4. Correlations between Levels of Arginine Derivatives in the Analyzed Groups

**Figure 1:**
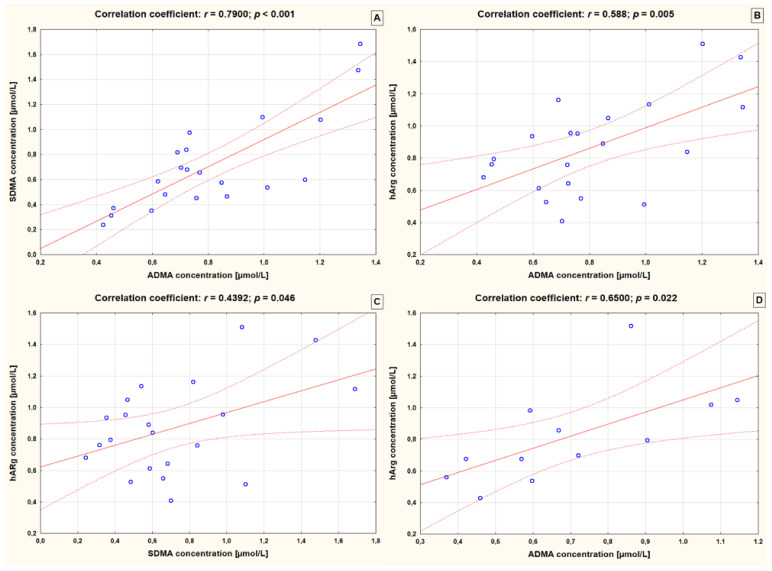
Significant correlations between arginine derivatives in controls (**A**–**C**) and epileptic children receiving 2 AEDs (**D**). ADMA—asymmetric dimethylarginine; SDMA—symmetric dimethylarginine; hArg—homoarginine; r—Pearson’s correlation coefficient.

Significant correlation coefficients between arginine derivatives in both controls and epileptic children are presented in Figure 1. In children with epilepsy who were taking two AEDs, only levels of hArg correlated positively with ADMA levels (r = 0.650, *p* = 0.022). The higher the level of hArg the greater the level of ADMA on average. A similar correlation was found in controls. In addition, children without epilepsy presented positive correlations between hArg and SDMA, as well as between ADMA and SDMA (r = 0.439, *p* = 0.046 and r = 0.790, *p* < 0.001, respectively).

## 4. Discussion

To the best of the authors’ knowledge, the present study is the first study regarding the serum concentrations of SDMA and hArg in the pediatric patients with epilepsy. No differences in mean concentrations of ADMA and SDMA were found between the whole study groups. However, in epilepsy patients, we observed a trend towards lower hArg levels compared to the control group. It was a difference close to the limit of significance. This finding may suggest that epileptic children may be at higher risk for CVD in the future. The results of the studies based on adults raised such a relation [11]. The study by Atzler et al. [16], based on a large cohort of adult patients from the US, demonstrated an inverse and independent correlation between hArg concentrations and aortic wall thickness after adjustment for several traditional CVD risk factors. On contrary, no correlation was observed between hArg and aortic plaque burden or coronary artery calcium [17].

In our study, the levels of analyzed parameters did not differ between girls and boys from the reference group. In the group of epileptic children, no relation was also observed for ADMA and hArg. However, the mean serum concentration of SDMA was slightly higher in girls with epilepsy than in boys but again insignificant.

As we analyzed epileptic children receiving two or more AEDs, we divided this group into two subgroups depending on the AEDs number, i.e., two AEDs and three or more AEDs. We observed that epileptic children receiving three or more AEDs presented significantly lower levels of hArg than controls. In turn, there was a tendency for a lower mean level of hArg in children receiving three or more AEDs compared to children receiving two AEDs. This may indicate that the higher the number of AEDs taken, the lower the hArg level.

The elevated plasma level of ADMA in the patients with atherosclerosis is related to the degree of dysfunction of vascular endothelium as well as the severity of the disease [18]. ADMA also increases oxidative stress and monocyte adhesion. Epileptic children receiving VPA were characterized by a simultaneous increase in ADMA and HCys concentrations compared to controls [7]. The latest research by Mahmoud et al. [19] found that long-term use of AEDs can increase lipid profiles, homocysteine, and ADMA levels. The authors also observed that this effect is particularly noticeable in the case of polytherapy with old-generation drugs, while in the case of new AEDs, a minimal effect was observed. [19].

In turn, L-homoarginine can regulate NO release. Similar to L-arginine, hArg is known as a vasodilator. The role of NO in the development of epileptic seizures is controversial. However, the synthesis of NO plays a role in the neural control of cerebral circulation [20]. Expression of neuronal NOS (nNOS) is also observed in peripheral autonomic nerves which modulate vessel tone [21]. When NO is overexpressed, its neurotoxicity is observed. In turn, dysfunctional nNOS signaling may be related to CVD events, e.g., brain ischemia and migraine [22,23]. Previously, it was observed that nNO production appears to exacerbate acute ischemia, while vascular NO protects after middle cerebral artery occlusion [22]. Few research on the relationship of hArg level with epilepsy have been conducted among adult patients but none in children. The study by Shiraga et al. [24] demonstrated significantly lower concentrations of hArg in both men and women suffering from epilepsy who received VPA compared to patients who were not treated with VPA. In addition, the authors observed also lower serum concentrations of hArg in women with uncontrolled epilepsy than in women with controlled epilepsy [24].

In the present study, we have also analyzed ratios of arginine derivatives. We observed that epileptic children receiving three or more AEDs had a significantly lower mean value of hArg/ADMA ratio than controls. Previously, the low hArg/ADMA ratio was demonstrated as an independent factor associated with higher cardiovascular mortality as well as a higher incidence of cardiovascular events in claudicant patients with lower extremity arterial disease [25]. The authors found also a similar association for hArg/SDMA ratio [25]. On contrary, our study demonstrated the higher mean value of hArg/ADMA ratio in children receiving two AEDs compared to children receiving three or more AEDs. In the whole study groups, the above-mentioned ratios were comparable between epileptic children and reference group. We also observed that there was a positive correlation between the levels of hArg and ADMA in children with epilepsy taking two AEDs. With an increase in the average concentration of hArg, the level of ADMA increases on average (r = 0.650, *p* = 0.022).

Several limitations can be pointed out for the present study. The most important one is the low number of analyzed patients. This aspect affects the statistics and thus the conclusions should be drawn with caution. Moreover, the reference group of children epilepsy-free was recruited in the neurology ward among children who were hospitalized with mild to moderate head injuries. As previously reported, this traumatic brain injury may result in significant temporal changes in ADMA and its metabolizing enzymes [26]. In the present study, we did not analyze epileptic children treated with monotherapy as cases with epilepsy suffered also from other neurologic disorders, which may influence the control of the seizures with a single AED. In addition, from the beginning, the project was planned to assess the effect of AED polytherapy on CVD risk factors since the effect of monotherapy is better known.

## 5. Conclusions

Epileptic children analyzed in the present study showed a tendency to a lower level of hArg compared to controls. In addition, after dividing patients depending on the number of AEDs we observed that taking three or more AEDs by epileptic children resulted in lower levels of hArg and a value of hArg/ADMA ratio. This finding may suggest that epileptic children may be at higher risk for CVD in the future. However, due to the low number of analyzed individuals (both in patient and reference groups), further research is needed to confirm our findings.

Our study is the first but preliminary research regarding the relationship between multi-drug therapy with AEDs and concentrations of SDMA and hArg in epileptic children. The knowledge obtained in this study may be the starting point for further research on the optimization of epilepsy therapy in order to reduce the influence of biochemical parameters on the endothelial wall or the processes leading to atherosclerosis in adulthood. In the future, we plan to expand the research we have started with the following aspects: enlargement of the study group, unification of the group of patients with the same AEDs, an extension of the cardiovascular assessment time of children receiving AEDs, and the use of non-invasive techniques to assess the condition of blood vessels, e.g., intima-media thickness.

## Figures and Tables

**Table 1:** General characteristics of the analyzed groups of patients.

Variable	Epileptic Patients *N* = 21	Reference Group *N* = 22	*p*
Age of the patient (years), M ± SD	7.13 ± 4.40	7.73 ± 3.00	0.522
Sex (F/M)	8/13	7/15	0.755
Age at epilepsy onset (years), M ± SD	2.46 ± 2.40	—	—
Duration of the treatment with AEDs (years), M ± SD Median, min.—max.	4.60 ± 4.28 2.75 (0.50–15.50)	—	—

M—mean; SD—standard deviation; F–female; M–male.

**Table 2:** The frequencies of AEDs combinations and types of seizures in analyzed children with epilepsy.

Combination of AEDs	*N* (%)	Morphology of Seizures	*N* (%)
VPA, LEV	6 (28.57)	generalized seizures	14 (66.67)
VPA, CLB	2 (9.52)	tonic-clonic seizures	2 (9.52)
VPA, VGB	1 (4.76)	polymorphic seizures	5 (23.81)
LEV, LTG	2 (9.52)	myoclonic	5 (23.81)
LEV, TPM	1 (4.76)	consciousness disturbances	4 (19.05)
LEV, PHB	1 (4.76)	tonic seizures	2 (9.52)
VPA, LEV, CLB	1 (4.76)	atonic seizures	1 (4.76)
VPA, LEV, CZP	1 (4.76)	focal aware seizures	2 (9.52)
VPA, TPM, VGB	1 (4.76)		
CLB, LTG, LEV	1 (4.76)		
PHB, LTG, LEV	1 (4.76)		
LEV, CZP, LTG	1 (4.76)		
CZP, LTG, TPM	1 (4.76)		
VPA, CLB, STP, LEV	1 (4.76)		

VPA—valproate; LEV—levetiracetam; CLB—clobazam; VGB—vigabatrin; LTG—lamotrigine; TPM—topiramate; PHB—phenobarbital; CZP—clonazepam; STP—stiripentol.

**Table 3:** Levels of arginine derivatives in all the analyzed groups.

Arginine Derivatives	Epileptic Children *N* = 21	Reference Group *N* = 22	*p*
ADMA (µmol/L), M ± SD	0.81 ± 0.37	0.82 ± 0.27	0.353
SDMA (µmol/L), M ± SD	0.64 ± 0.21	0.72 ± 0.36	0.933
hArg (µmol/L), M ± SD	0.72 ± 0.29	0.86 ± 0.29	0.091
ADMA/SDMA, M ± SD	1.40 ± 0.86	1.27 ± 0.38	0.857
hArg/ADMA, M ± SD	0.99 ± 0.40	1.11 ± 0.37	0.283
hArg/SDMA, M ± SD	1.29 ± 0.68	1.44 ± 0.71	0.463

M—mean; SD—standard deviation; ADMA— asymmetric dimethylarginine; SDMA— symmetric dimethylarginine; hArg—homoarginine. *p* for Mann–Whitney *U* test.

**Table 4:** Levels of analyzed arginine derivatives in subgroups depending to the number of AEDs.

Arginine Derivatives	Epileptic Children Receiving 2 AEDs *N* = 13	Epileptic Children Receiving 3 or More AEDs *N* = 8	Reference Group *N* = 22	*p*
ADMA (µmol/L), M ± SD	0.69 ± 0.24	1.00 ± 0.47	0.82 ± 0.27	0.219
SDMA (µmol/L), M ± SD	0.61 ± 0.21	0.69 ± 0.23	0.72 ± 0.36	0.845
hArg (µmol/L), M ± SD	0.79 ± 0.30	0.60 ± 0.24	0.86 ± 0.29	**0.059**
ADMA/SDMA, M ± SD	1.21 ± 0.40	1.71 ± 1.29	1.27 ± 0.38	0.865
hArg/ADMA, M ± SD	1.18 ± 0.35	0.67 ± 0.25	1.11 ± 0.37	**0.005**
hArg/SDMA, M ± SD	1.42 ± 0.58	1.07 ± 0.81	1.44 ± 0.71	0.204

M—mean; SD—standard deviation; AED—antiepileptic drugs; ADMA— asymmetric dimethylarginine; SDMA— symmetric dimethylarginine; hArg—homoarginine; *p* for Kruskal–Wallis test. Significant differences are in bold.

## Data Availability

The data presented in this study are available on request in the Department of Basic Biomedical Science, Faculty of Pharmaceutical Sciences, Medical University of Silesia in Katowice (Poland). The data are not publicly available due to privacy restrictions.
